# The Function of Membrane Integral Pyrophosphatases From Whole Organism to Single Molecule

**DOI:** 10.3389/fmolb.2019.00132

**Published:** 2019-11-22

**Authors:** Alexandra O. M. Holmes, Antreas C. Kalli, Adrian Goldman

**Affiliations:** ^1^School of Biomedical Sciences and Astbury Centre for Structural Molecular Biology, University of Leeds, Leeds, United Kingdom; ^2^Leeds Institute of Cardiovascular and Metabolic Medicine and Astbury Centre for Structural Biology, University of Leeds, Leeds, United Kingdom; ^3^Research Program in Molecular and Integrative Biosciences, University of Helsinki, Helsinki, Finland

**Keywords:** membrane-integral pyrophosphatases, human pathogens, plants, structural biology, molecular mechanism, membrane proteins, hydrolysis, ion pumping

## Abstract

Membrane integral pyrophosphatases (mPPases) are responsible for the hydrolysis of pyrophosphate. This enzymatic mechanism is coupled to the pumping of H^+^ or Na^+^ across membranes in a process that can be K^+^ dependent or independent. Understanding the movements and dynamics throughout the mPPase catalytic cycle is important, as this knowledge is essential for improving or impeding protein function. mPPases have been shown to play a crucial role in plant maturation and abiotic stress tolerance, and so have the potential to be engineered to improve plant survival, with implications for global food security. mPPases are also selectively toxic drug targets, which could be pharmacologically modulated to reduce the virulence of common human pathogens. The last few years have seen the publication of many new insights into the function and structure of mPPases. In particular, there is a new body of evidence that the catalytic cycle is more complex than originally proposed. There are structural and functional data supporting a mechanism involving half-of-the-sites reactivity, inter-subunit communication, and exit channel motions. A more advanced and in-depth understanding of mPPases has begun to be uncovered, leaving the field of research with multiple interesting avenues for further exploration and investigation.

## Introduction

Pyrophosphatases (PPases) are enzymes responsible for the reversible hydrolysis of the phosphoanhydride bond in pyrophosphate (PP_i_) to two inorganic phosphate molecules (Kajander et al., 2013). PPases are subcategorized into three separate protein families; Family I, Family II, and membrane-integral pyrophosphatases (mPPases). Families I and II are evolutionarily unrelated soluble proteins. Despite their common enzymatic activity, mPPases are vastly different to the other PPase families. Firstly, the architecture and sequence of these protein families are unrelated. Ignoring the oligomeric structure, a single subunit of both Family I and mPPases consists of a single domain, while Family II PPases have two domains per subunit. mPPases are the only family that is embedded in the membrane: they have 15-17 transmembrane helices (TMH) per 70-81 kDa subunit (Kellosalo et al., 2012; Lin et al., 2012). Secondly, mPPases have only been reported as homodimers, whereas Family I PPases can form other oligomeric arrangements (Kankare et al., 1996; Kellosalo et al., 2012; Lin et al., 2012; Li et al., 2016; Tsai et al., 2019; Vidilaseris et al., 2019). Family I and II PPases are only responsible for removing excess waste PP_i_ from the cytoplasm, while mPPases are primary ion pumps: they couple hydrolysis to movement of H^+^ and/or Na^+^ across a membrane (Kajander et al., 2013) and thus generate a membrane potential, which contributes to a number of cellular functions, such as acidocalcisome and vacuole regulation and energization (Shah et al., 2016). The catalytic activities of these protein families differ by orders of magnitude. The mPPases are the slowest as they can only hydrolyze ≈ 10 PP_i_ molecules per second, followed by Family I, which can hydrolyze ≈ 200 molecules per second, and Family II which can hydrolyze ≈ 2,000 per second (Kajander et al., 2013).

mPPases are less abundant than soluble PPases but still occur in all kingdoms of life except fungi and multicellular animals (Kajander et al., 2013). In eukaryotes, they are localized in the membranes of organelles, such as the Golgi apparatus (Mitsuda et al., 2001), plant vacuoles (Gaxiola et al., 2007), or the acidocalcisomes of protists (Moreno and Docampo, 2009). They are also present in the inner cellular membrane of bacteria such as *Bacteroides vulgatus* (Luoto et al., 2013a). mPPases have been found to play a role in stress tolerance and plant maturation. Finally, mPPases promote energy efficiency and survival in numerous human pathogens, making them clinically relevant as potential drug targets (Luoto et al., 2011; Shah et al., 2016).

This evolutionarily ancient family evolved through a gene triplication; they thus consist of three structurally-conserved splayed 4-helix bundles made up of TMH 3-6, 9-12, and 13-16 (Au et al., 2006; Kajander et al., 2013) arranged with ~3-fold symmetry perpendicular to the membrane plane. The bundles are structurally highly similar with RMSD/C_α_ values of 2.1–2.9 Å between them (Kellosalo et al., 2012) and also have 23.6–26.1% sequence identity (Kellosalo et al., 2012). Phylogenic analysis suggests that hydrolysis of PP_i_ to provide energy may have occurred prior to the adoption of ATP as the universal energy currency (Baltscheffsky et al., 1999). Therefore, mPPases may have been the first enzymes to couple phosphoanhydride bond formation/hydrolysis to chemiosmotic potential. Since the initial discovery of mPPase in *Rhodospirillum rubrum*, seven different mPPase subfamilies have been discovered (Table 1) (Luoto et al., 2011, 2015; Tsai et al., 2014). In brief, the different mPPases are subdivided into two main groups: (i) K^+^ independent, which pump protons (H^+^-PPases) and can be regulated by Na^+^, and (ii) K^+^ dependent, which can function in the absence of K^+^, but require K^+^ for maximal activity. Of these, there are H^+^-PPases, Na^+^ pumping (Na^+^-PPase), and dual Na^+^/H^+^ pumping (Na^+^/H^+^-PPase) PPases (Kajander et al., 2013). Sequence analysis suggests that the first mPPases were Na^+^-PPases, and that H^+^-PPases evolved from these four independent times (Baykov et al., 2013). Additionally, it is likely that the evolution of Na^+^/H^+^-PPases occurred separately to the H^+^-PPases (Luoto et al., 2013b).

**Table 1:** mPPase subfamily classification.

**Cation Pumping Specificity**	**Monovalent Cation Dependence**	**Semi-Conserved Glutamate Location**	**Regulation**	**Hypothesized Sodium Binding Sites**	**Example**	**References**
H^+^	K^+^	6.57	–	–	*Vigna radiata*	Tsai et al., 2014
		6.53	–	–	*Carboxydothermus hydrogenoformans*	Tsai et al., 2014
		5.43	–	–	*Flavobacterium johnsoniae*	Tsai et al., 2014
	-	6.53	Na^+^ and K^+^	Inhibitory	*Chlorobium limicola*	Luoto et al., 2015
			–	–	*Pyrobaculum aerophilum*	Luoto et al., 2011
Na^+^	K^+^ and Na^+^	6.53	–	Activating and Inhibitory	*Thermotoga maritima*	Tsai et al., 2014
Na^+^ and H^+^	K^+^ and Na^+^	6.53	–	Activating	*Bacteroides vulgatus*	Tsai et al., 2014

This review encompasses our current knowledge of mPPase function, including their evolution, role in whole organisms, and their structure and mechanism on the molecular level. We also suggest avenues for future exploration.

## Function and Relevance

### Plants

Under physiological conditions in plants, H^+^-PPases are predominantly localized to the tonoplast membrane surrounding the vacuole (Segami et al., 2014) and make up 10% of its protein components (Segami et al., 2018a). The vacuole possesses multiple functions that require the large-scale movement of molecules across its membrane. The required membrane potential is generated by the vacuolar ATPase complex (V-ATPase) in combination with H^+^-PPases (Kriegel et al., 2015). There is some controversy over the delineation of the roles of these proton pumps, as a V-ATPase knock-out strain was able to properly maintain acidification of the vacuole and normal function (Krebs et al., 2010). However, a more recent study suggested that lack of V-ATPase could not be compensated for by increased mPPase activity (Kriegel et al., 2015). The general consensus is that the role of H^+^-PPase in stress tolerance is to replace V-ATPase activity when ATP levels are low (Maeshima, 2000), but this does not fully address the roles of the proton pumps under normal conditions.

H^+^-PPases are important in plant maturation (Li et al., 2005) because they remove PP_i_ from the cytoplasm (Ferjani et al., 2011; Asaoka et al., 2016). PP_i_ is the by-product of many different cellular processes, including the biosynthesis of protein, RNA and, importantly for plants, cellulose (Maeshima, 2000). Removing the excess PP_i_ following these reactions is critical for driving these processes. Additionally, PP_i_ has a modulatory role as a biochemical intermediate of a number of enzymes (Heinonen, 2001), so tight control of its cytoplasmic availability is essential for normal cellular function. mPPase over-expression in *Arabidopsis thaliana* resulted in increased cell division and hyperplasia of different organs, in particular the leaves. In contrast, knock-out mutants, and RNA interference studies showed severely disrupted root and shoot development. Each of these were linked to increased or decreased trafficking of the phytohormone auxin, which is known to mediate organogenesis (Li et al., 2005), suggesting a role for H^+^-PPases in auxin regulation.

This role in auxin regulation was further highlighted in studies of transgenic plants over-expressing H^+^-PPase genes. Multiple studies have shown that increased polar auxin transport upon mPPase over-expression is closely related to improved root development under stress conditions (Li et al., 2005; Park et al., 2005; Pasapula et al., 2011; Zhang et al., 2011). This plays a role in drought resistance, as the larger root system provides enhanced water absorption (Zhang et al., 2011). In addition to the increased root biomass mechanism, the effect of H^+^-PPase over-expression on vacuolar function improved tolerance of drought and salinity. The increased electrochemical gradient may drive uptake of ions into the vacuole, producing an increase in osmotic potential and stimulating water uptake (Park et al., 2005; Brini et al., 2006; Zhao et al., 2006; Pasapula et al., 2011; Zhang et al., 2011). Further evidence of the potential of mPPases to improve crop tolerance to suboptimal conditions were the reports of increased chlorophyll content, photosynthesis, leaf water content and fiber yield, with decreased cell membrane damage in transgenic cotton plants, as compared to wild-type under low water and high salt conditions (Lv et al., 2009; Pasapula et al., 2011). Interestingly, these effects may not be predominantly due to the proton pumping activity of the vacuolar mPPases. One study saw a *Vr*-PPase mutant lacking proton pumping activity, but retaining hydrolysis, was sufficient to rescue the stunted knock-out phenotypes (Asaoka et al., 2016). In addition to this, an *A. thaliana* plant H^+^-PPase knockout saw no heterotrophic growth following germination, but this phenotype was rescued by soluble PPase expression, suggesting that effective PP_i_ clearance is the primary function of H^+^-PPases during postgerminative growth in *Planta* (Ferjani et al., 2011). It is not clear why this is the case, as all plant cells express soluble Family I PPases at concentrations that should be sufficient to clear the pyrophosphate generated. An explanation could be that the soluble and mPPases function cooperatively (Segami et al., 2018b). In this model, the H^+^-PPase functions as the major cytosolic PP_i_-hydrolysis enzyme and the soluble PPases contribute to preventing accumulation to toxic levels, which would explain how soluble PPase expression was able to somewhat compensate for mPPase loss in the aforementioned study (Ferjani et al., 2011).

### Human Pathogens

#### Protozoan Pathogens

A number of major human diseases are caused by protozoan parasites, for example, malaria (*Plasmodium ssp*.), toxoplasmosis (*Toxoplasma gondii*), trypanosomiasis (*Trypanosome spp*.), and leishmaniasis (*Leishmania spp*.) (Shah et al., 2016). These diseases each have a high prevalence and risk of fatality (Büscher et al., 2017; World Health Organization, 2018) or association with other diseases. For example, toxoplasmosis has been suggested to be associated with a number of conditions, such as psychiatric, neurological, and neoplastic disorders (Torgerson and Mastroiacovo, 2013; Flegr et al., 2014). In addition to this, several protozoan strains responsible for malaria and trypanosomiasis have emerged that are resistant to most of the current treatment regimes (Büscher et al., 2017; World Health Organization, 2018). Therefore, there is a demand for novel therapeutics for these tropical diseases.

The protozoan parasite life cycle typically involves transitions between vectors and hosts and intracellular to extracellular environments, which means the protozoan cell must survive and adapt to several different conditions (Crompton et al., 2014). In terms of mPPase function, the most relevant change to overcome is the difference in osmotic pressure the cells experience in these different environments. The main protozoan mechanism for adjusting internal osmotic pressure involves the acidocalcisome (Docampo et al., 2013), where mPPases are localized in protozoa (Scott et al., 1998; Marchesini et al., 2000). This is a small acidic compartment where numerous ions are stored, including polyphosphate, which the parasite hydrolyses or synthesizes in response to osmotic stress and to release energy (Ruiz et al., 2001). The low pH of the acidocalcisome is crucial for its function, as loss of acidity can lead to a 10-fold decrease in stored polyphosphate levels (Lemercier et al., 2002), resulting in reduced capability to respond to osmotic changes. In addition to the effects on polyphosphate storage, there are detrimental effects on intracellular pH regulation, growth rate and final cell density (Lemercier et al., 2002), suggesting that the loss of mPPases has more widespread effects than just reduced osmotic regulation.

As mPPase function in protozoa is highly important, mPPases are a validated target for pharmacological intervention. Knock-down and knock-out studies in *Trypanosoma brucei* (Lemercier et al., 2002) *T. gondii* (Liu et al., 2014), and *P. falciparum* (Zhang et al., 2018) have demonstrated that mPPases are required for maintaining acidocalcisome acidification, parasitic virulence and *in vitro* asexual bloodstage growth. Additionally, mPPase-inhibiting bisphosphonate derivatives retarded intracellular proliferation of *T. gondii* with no effect on host cells (Rodrigues et al., 2000).

#### Bacterial Pathogens

The most relevant mPPase-expressing bacterial genus to human health are *Bacteroides spp*., especially *B. vulgatus* and *Bacteroides fragilis* (Luoto et al., 2013a), which form a mutualistic relationship with healthy individuals as part of the gastrointestinal microflora (Wexler, 2007). However, outside this environment, they can cause bacteremia (presence of bacteria in the blood), intra-abdominal sepsis, appendicitis, gynecological, and skin infections, endocarditis, septic arthritis, and abscesses in tissues including the brain and female urogenital tract (Wexler, 2007). This is a major threat to human health, as *Bacteroides* reportedly have the highest resistance rates of all anaerobic pathogens (Wexler, 2007), and an associated mortality rate of over 19%, rising to 60% in untreated cases (Goldstein, 1996).

Bacterial mPPases are primarily found in species that exist under conditions of low-energy stress, such as the obligate anaerobes *Bacteroides* and deep-sea organisms (*Thermotoga maritima* and *Pyrobaculum aerophilum*) (Luoto et al., 2011). In these situations, mPPases may be essential to increasing energy efficiency and promoting survival. mPPases have also been shown to confer greater resistance to other stress conditions. One study revealed that expression of transgenic plant mPPases in *Escherichia coli* and *Saccharomyces cerevisiae* resulted in improved tolerance to heat, hydrogen peroxide and high salinity (Yoon et al., 2013). Therefore, we speculate that inhibiting mPPase activity could have effects on bacterial viability in response to stressors, similar to the effects seen in protozoan parasites and plants.

## Structure and Mechanism

### Structural Overview

**Figure 1:**
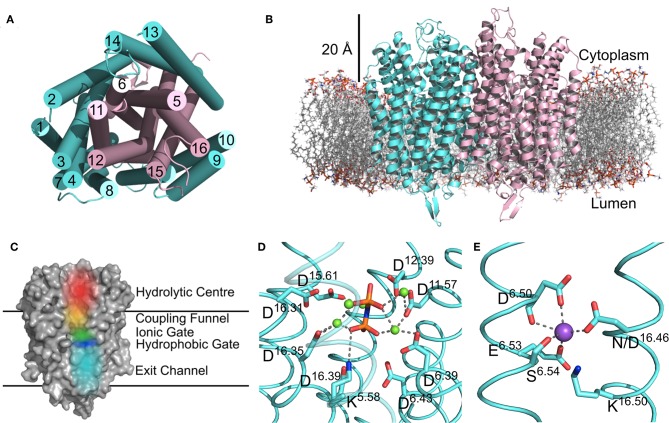
Structural Features of mPPases. **(A)** The concentric ring arrangement of the 16 helices in one mPPase subunit, as viewed from the cytoplasmic side. **(B)** The dimeric protein and its positioning in the lipid bilayer viewed from the membrane plane. The mPPase subunits are colored cyan and pink. **(C)** A surface view of one mPPase subunit with each of the catalytic regions colored (red, hydrolytic center; yellow, coupling funnel; green, ionic gate; dark blue, hydrophobic gate; light blue, exit channel). The coordination of **(D)** IDP and Mg^2+^ (green spheres) in the hydrolytic center and **(E)** of Na^+^ (purple sphere) at the ionic gate. Figures generated using PyMol (DeLano, 2002) and the IDP-bound *Tm-*PPase structure (PBD: 5LZQ) (Li et al., 2016).

There are now several structures of mPPases (Table 2). In the current structures, each subunit of the homodimeric mPPase is formed by 16 TMH, which form two concentric rings (Figure 1A) surrounding the four catalytic regions: the hydrolytic center, the coupling funnel, the ion gate and the exit channel (Figure 1C) (Kellosalo et al., 2012; Lin et al., 2012; Li et al., 2016). The protein subunit-subunit interface is maintained by hydrophobic interactions and hydrogen bonds between residues on TMH 10 (I/R^10.33^,N^10.42^, S^10.45^, K^10.49^), 13 (L^13.19^, L/V^13.21^, N^13.22^, M/V^13.23^, I^13.33^, Y^13.40^, S^13.48^, G^13.54^, E^13.59^, R^13.62^), and 15 (S^15.45^, Q^15.48^) (Shah et al., 2016). Here, we use the Ballesteros and Weinstein numbering system, first introduced for GPCRs, so that all functional residues have the same index (Residue^helixnumber.offset^) (Ballesteros and Weinstein, 1995), and which we adopted for mPPases (Tsai et al., 2014). In this system, each residue is assigned two numbers, one indicating the TMH and the other to describe its offset from a well-conserved residue close to the middle of the TMH (assigned position 50), as shown in Table 3. For example, K415 from *Tm*-PPase, is on TMH10 and is one before the conserved S416 and is thus designated K^10.49^.

**Table 2:** Published mPPase structures.

**Species**	**Ligand^**a**^**	**Ions^**a**^**	**Conformation**	**Mutation**	**PDB**	**Resolution (Å)**	**Noteworthy**	**References**
*Vigna radiata*	IDP	5 Mg^2+^ K^+^	Symmetrical IDP-Bound	–	4A01	2.35	First mPPase Structure	Lin et al., 2012
	PO_4_	2 Mg^2+^	Relaxed Product Bound	–	5GPJ	3.5		Li et al., 2016
	2 PO_4_	5 Mg^2+^ K^+^	Product Bound	–	6AFS	2.3	Demonstrated Exit Channel Width Affects Function	Tsai et al., 2019
				E^6.57^Q	6AFT	2.5		
				L^12.64^M	6AFU	2.8		
				L^12.64^K	6AFV	2.7		
				T^5.36^D	6AFW	2.2		
				E^5.33^A	6AFX	2.3		
				E^5.33^S	6AFY	2.4		
				E^5.33^H	6AFZ	2.5		
*Thermotoga maritima*	-	Mg^2+^ Ca^2+^	Resting	–	4AV3	2.6		Kellosalo et al., 2012
	2 PO_4_	4 Mg^2+^ K^+^	Product Bound	–	4AV6	4.0		Kellosalo et al., 2012
	IDP	5 Mg^2+^ Na^+^	Symmetrical IDP-Bound	–	5LZQ	3.5		Li et al., 2016
	WO_4_	2 Mg^2+^	Relaxed Product Bound	–	5LZR	4.0		Li et al., 2016
	2 ATC^b^ IDP	5 Mg^2+^ Na^+^	Locked	–	6QXA	3.7	Asymmetrical Structure Allosteric Inhibitor	Vidilaseris et al., 2019

a *The number of ligands or ions per subunit*.

b *Only bound to one of the subunits*.

*IDP, imidodiphosphate; ATC, N-[(2-amino-6-benzthiazolyl)methlyl]-1H-indole-2-carboxamide*.

**Table 3:** Ballesteros and weinstein numbering for mPPases.

**TMH**	**Signifier**	**% Sequence Identity**	**Residue in *Tm*-PPase**	**Residue in *Vr-*PPase**	**Residue in *Pa-*PPase**
1	1.50	38.1	F17	F25	Y20
2	2.50	27.6	K55	K94	R58
3	3.50	99.5	S87	S153	S96
4	4.50	92.9	G130	G194	G138
5	5.50	86.4	R191	R242	R182
5-6 Border^a^	5.77	99.9	D218	D269	D210
5-6 Border^b^	6.26	96.2	D219	D270	D211
6	6.50	99.7	D243	D294	D235
7	7.50	46.3	G297	G334	A271
8	8.50	75.8	L321	L361	L296
9	9.50	91.1	G369	G411	G333
10	10.50	84.3	S416	S458	S380
11	11.50	98.1	D458	D500	D439
12	12.50	99.7	K499	K541	K480
13	13.50	99.4	V566	V597	V554
14	14.50	79	M611	M642	F599
15	15.50	94	A649	A680	S637
16	16.50	94.5	K707	K742	K691

a *The last TMH5 residue*.

b *The first TMH6 residue*.

The inner ring is composed of six TMH (Baltscheffsky et al., 1999; Moreno and Docampo, 2009; Luoto et al., 2013a; Tsai et al., 2014, 2019; Vidilaseris et al., 2019) and the outer ring is composed of the remaining 10 (Figure 1A) (Kellosalo et al., 2012; Lin et al., 2012; Li et al., 2016). A large hydrophilic region extends about 20 Å into the cytoplasm (Figure 1B). This region has a hydrolytic center, which is lined with highly conserved negative (D^5.61^, D^5.65^, E^5.76^, D^5.77^, D^6.35^, D^6.39^, D^6.43^, D^11.57^, D^15.61^, D^16.31^, D^16.35^, D^16.39^), positive (K^5.58^, K^15.64^, K^16.38^) and polar (N^12.53^) residues that are involved in substrate binding through both direct interactions and *via* coordination of Mg^2+^ and water (Figure 1D). The hydrolytic center is followed by the coupling funnel, which is comprised of an ionic network between TMH 5-6 (R^5.50^, K^5.58^, D^6.39^, D^6.50^), 11-12 (D^11.50^, K^12.50^) and 16 (K^16.38^, D^16.39^). Below this is the ionic gate, which is situated in the center of the membrane and where the cation is bound prior to pumping (Figure 1E). This region is made up of charged and hydrophilic residues (S^5.43^, D^6.50^, E/S^6.53^, S^6.54^, E^6.57^, D^16.46^, K^16.50^). The hydrophobic gate located near the ionic gate and is formed by semi-conserved non-polar residues on TMH 5, 6, 12, and 16. Mutations of residues near the ionic and hydrophobic gates (I^12.54^, L^12.64^, D^16.46^, V^16.54^, L^16.57^) to alanine decouple the enzyme so that mPPase can hydrolyze PP_i_ but does not pump, suggesting these regions are also essential for the coupling of hydrolysis to pumping (Asaoka et al., 2014). Finally, the residues of the exit channel are not highly conserved between mPPases. However, allosteric inhibitor binding to this region in one *Tm*-PPase subunit is nonetheless associated with a “locked” conformation (Vidilaseris et al., 2019) and exit channel width has been implicated in coupling hydrolysis and pumping (Tsai et al., 2019), thereby illustrating the importance of conformational changes in this region during the catalytic cycle.

### Structural Differences Between Subfamilies

#### Cation Specificity

Mutagenesis and structural studies showed that the residues E^6.53/6.57^, S^6.54^, and D/N^16.46^ are involved in cation binding and pumping specificity (Asaoka et al., 2014; Li et al., 2016). The main residue regulating cation specificity is the semi-conserved glutamate on TMH6. Movement of this glutamate down one helical turn from E^6.53^ to E^6.57^ converts a Na^+^-PPase to a H^+^-PPase by destroying the Na^+^ binding site in the substrate bound state (Li et al., 2016). However, the reverse mutation does not yield a Na^+^-PPase, indicating that the factors dictating cation specificity are more complex than solely the location of a single residue.

#### K^+^ Dependence and Independence

Mutation of A^12.46^ to lysine in the active site converts a K^+^-dependent enzyme to an independent enzyme (Belogurov and Lahti, 2002). Modeling this mutation suggested that the ${\text{NH}}_{3}^{+}$ group of the lysine sidechain functionally replaces the K^+^ ion at physiological PP_i_ concentrations (Kellosalo et al., 2012; Lin et al., 2012). A systematic analysis of the potassium ion binding site or active site lysine (K^+^/K^12.46^ catalytic center) across all mPPase subfamilies revealed that the lysine contributed to, but was not essential, for the activity of K^+^ independent mPPases, as the activity was restored at high K^+^ concentrations when mutated (Artukka et al., 2018). This suggests that K^12.46^ masks a K^+^ binding site, but there appear to be larger-scale differences between the two classes than just the identity of A/K^12.46^ in the active site (see Inter-subunit Communication).

#### Na^+^ Regulatory Sites

The difference between Na^+^- or H^+^-PPases and Na^+^/H^+^-PPases was unclear prior to the discovery of Na^+^ and K^+^ regulated H^+^-PPases (Luoto et al., 2015). These are a subfamily of K^+^ independent H^+^-PPases, in which low K^+^ concentrations enhance H^+^ transport but higher Na^+^ and K^+^ concentrations inhibit ion pumping and hydrolysis. This led to the hypothesis that there are two possible Na^+^ binding sites; one in the ion conductance channel associated with activation of the mPPase, and the other located away from the conductance channel conferring inhibition of H^+^ conductance, to which K^+^ can bind with low affinity. This fits with the reports of H^+^ translocation by Na^+^-PPases in sub-physiological sodium concentrations (Luoto et al., 2013a), suggesting that loss of the second inhibitory site led to the evolution of the Na^+^/H^+^-PPases.

#### Mechanism of Pumping and Hydrolysis

Three mechanisms have been proposed for mPPase pumping and hydrolysis: (i) pumping occurs upon binding of substrate and prior to hydrolysis in a “binding change” type mechanism (Kellosalo et al., 2012), (ii) the proton released upon hydrolysis triggers release of the ions at the ion gate *via* a Grotthus type mechanism (Lin et al., 2012), and (iii) the hydrolysis-generated proton is directly transported in a “direct-coupling” mechanism (Baykov et al., 2013). This first mechanism assumes that the nucleophilic proton may not be involved in pumping, whereas the second and third propose that the hydrolysis-generated proton is the one pumped, thereby assuming hydrolysis precedes cation-pumping. However, neither the second nor the third mechanisms account for Na^+^ transport (Kajander et al., 2013), except for potentially *via* a “billiard-type” model in the third, where the Na^+^ is pushed into the exit channel by the nucleophilic water proton (Baykov et al., 2013). In our opinion, models in which hydrolysis precedes cation pumping do not conform with the studies of non-hydrolysable substrate analogs [IDP and methylene diphosphonate (MEDP)] binding to *Vr*-PPase (Li et al., 2016; Shah et al., 2017) resulting in a single ion turnover event.

**Figure 2:**
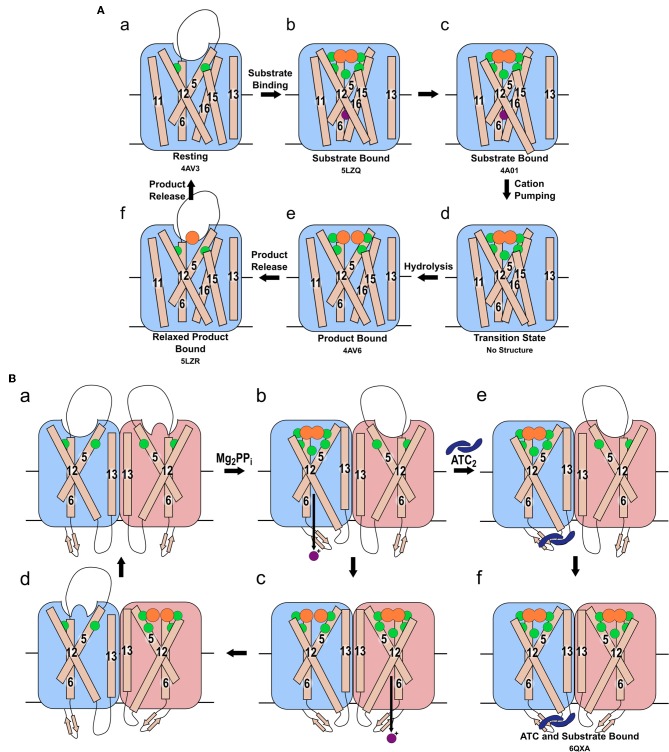
Mechanism of pumping and hydrolysis. **(A)** “Binding change” mechanism. In the resting state (a), a cation may be present, but its position has not been revealed structurally. When substrate binds, the hydrolytic center is occluded by movement of the 5-6 loop and of TMH 11-12 and 15-16, and a cation localizes to the ionic gate (b). The cation is then pumped (c) and TMH 12 resumes its original position (d). Hydrolysis then occurs (e). Prior to the sequential release of the product P_i_, the 5-6 loop, TMH 11-12 and 15-16 relax to their resting positions (f). **(B)** The proposed asymmetric cycle of the mPPase dimer. One site is capable of binding PP_i_, while the other is distorted (a). Upon binding of one PP_i_ molecule and ion pumping, the other site undergoes a conformational change, triggered by the movement of TMH13, to increase its affinity for PP_i_ (b). The second PP_i_ molecule binds and the second cation is pumped, while the first PP_i_ is hydrolyzed (c). Upon release of the resulting P_i_, this active site is distorted to reduce affinity for PP_i_ whilst the second bound PP_i_ undergoes hydrolysis (d). States (e,f): the proposed result of allosteric inhibitor ATC binding to the mPPase. The dimer is trapped in a state unable to hydrolyze PP_i_, but capable of binding one (e) or two (f) PP_i_. (Green spheres: Mg^2+^, purple spheres: cation for pumping, orange spheres: PP_i_ or PO_4_, blue dimeric shape: ATC). PDB IDs for structurally defined states are listed beneath the relevant image.

The “binding change” mechanism unifies both sodium and proton pumping, and is supported by studies indicating that charge transfer—and so presumably ion pumping—does not require hydrolysis (Li et al., 2016; Shah et al., 2017) and so presumably precedes it in the full catalytic cycle. In this model (Figure 2), the substrate binds to the hydrolytic center, which is then closed to the cytoplasm by ordering of the 5-6 loop and movement of TMHs 11-12 and 15-16 toward the center of the coupling funnel (Figure 2Aa,b). During these conformational changes, a cation is pumped out from the ionic gate (Figure 2Ac,d). The increase in overall negative charge in this region leads to downwards movement of TMH 12 by 2 Å and an associated bend of TMH11, as observed structurally (Figure 2Ab,c) (Kellosalo et al., 2012; Lin et al., 2012; Li et al., 2016). This causes deprotonation of the aspartate pair D^6.43^ and D^16.39^, which activates the water nucleophile leading to hydrolysis (Figure 2Ad,e) (Kajander et al., 2013). This process has been demonstrated to be independent of cation pumping in exit channel mutants (Asaoka et al., 2014). These mutations were initially proposed to alter the position and angle of their helices and thereby uncouple the reactions (Asaoka et al., 2014). However, more recent structural characterization of exit channel and hydrophobic gate mutants indicate that this uncoupling appear to be due to widening of the exit channel (Tsai et al., 2019), potentially allowing ion back-flow.

### Asymmetry and Inter-subunit Communication

The events occurring in each mPPase subunit during pumping and hydrolysis are relatively well-understood (see above), but recent studies have provided evidence of asymmetry and subunit interdependence. Firstly, the role of the K^+^/K^12.46^ catalytic center in inter-subunit communication, as inferred from the effects of excess substrate was published (Artukka et al., 2018) (see Inter-subunit Communication). Shortly after this, the first structural evidence of asymmetry, further functional evidence, and a putative mechanism involving both subunits were reported (Vidilaseris et al., 2019). The earliest reports of subunit interdependence were in the 1990s in a series of studies utilizing radiation-induced damage to identify the functional unit size of mPPases (Wu et al., 1991; Sarafian et al., 1992; Tzeng et al., 1996). Several of these showed that one impaired subunit conferred compromised function to the unaffected subunit (see Inter-subunit Communication).

#### Functional Asymmetry

Vidilaseris et al. (2019) reported that binding of the first substrate increased the affinity of the second subunit to PP_i_. They also observed that the binding of first substrate molecule potentiated binding of the second. This suggests *positive* co-operativity for substrate binding, and thus they proposed the following mechanism (Figure 2Ba): the first substrate binds to the first subunit leading to cation translocation in this subunit (Figure 2Bb), this causes conformational changes in the second subunit to optimize PP_i_ binding. Following the binding of the second substrate molecule, hydrolysis occurs in the first subunit and ion pumping in the second (Figure 2Bc). This then allows hydrolysis in the second subunit and product release (Figure 2Bd). However, the same study (Vidilaseris et al., 2019) also reported a 20-fold decrease in the maximal rate of hydrolysis when both subunits bind substrate, which does not appear to fit with their proposed asymmetric mechanism.

In contrast, Artukka et al. (2018) reported a *decrease* in affinity for PP_i_ at the second subunit following substrate binding to the first. The binding of fluorescein 5'-isothiocyanate (FITC) to a lysine believed to be in the catalytic center (K^12.50^) also decreases the affinity for FITC in the second subunit (Yang et al., 2000), providing further support for an allosteric mechanism. Binding at the first site thus presumably distorts the active site in the second subunit, reducing its affinity for PP_i_/FITC. Negative co-operativity for substrate binding is highly relevant, because excess substrate inhibits hydrolysis in all wild-type mesophilic mPPases studied so far (Artukka et al., 2018), so excess substrate may play a role in proton pumping inhibition (Luoto et al., 2015). As there is no evidence of an alternative PP_i_ binding site, and the hydrolytic center and coupling funnel could not accommodate further PP_i_ molecules, the inhibition must be a result of substrate binding to the second subunit. This would only be possible in excess substrate concentrations, due to the decreased affinity at the second site. Similar to Vidilaseris et al. (2019), this study also saw a 3–16-fold decrease in maximal velocity of hydrolysis in both subunits upon binding of the second substrate. This again supports the model in which excess substrate inhibits hydrolysis through binding at this second site, thus suggesting that at moderate PP_i_ concentrations only one subunit operates at any given time. The direct implication of this is that the symmetrical IDP bound structures are potentially not mechanistically relevant at typical PP_i_ concentrations, but a possible artifact of the 4 mM minimum IDP concentration during crystallization (Lin et al., 2012; Li et al., 2016). These papers by Artukka et al. (2018) and Vidilaseris et al. (2019) are the first two definitive studies of possible asymmetry: further data are needed to understand fully the mechanism involving both subunits.

What could explain the inconsistencies between these two studies? Firstly, one study investigated thermophilic protein in an assay at 71°C (Vidilaseris et al., 2019), whereas, the other used mesophilic protein at 30°C (Artukka et al., 2018). The behavior could indicate different properties between thermo- and mesophilic proteins, or different activities at different temperatures. Secondly, one study was performed with purified protein in detergent micelles (Vidilaseris et al., 2019) and the other used unpurified protein in inner membrane vesicles (Artukka et al., 2018). This could suggest that the lipid environment of the enzyme could be involved in regulating the dimeric interface, a concept for which there is increasing evidence (Gupta et al., 2017; Pyle et al., 2018).

#### Structural Asymmetry

**Figure 3:**
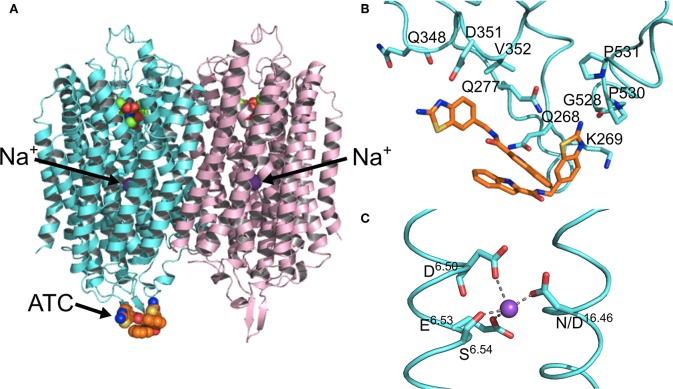
Structural asymmetry. **(A)** The location of the ATC molecule (orange spheres) and the Na^+^ ion (purple sphere) in the mPPase dimer. **(B)** The binding of the two ATC molecules (orange sticks) to the β1-2 loop, loop 8-9, and loop 12-13, with the interacting residues shown at sticks *(Tm*-PPase numbering). **(C)** The tetracoordination of Na^+^ (purple sphere) at the ionic gate. (PDB: 6QXA) (Vidilaseris et al., 2019).

Kinetic and structural studies showed that the non-phosphorous allosteric inhibitor *N*-[(2-amino-6-benzothiazolyl)methyl]-1*H*-indole-2-carboxamide (ATC) binds as a dimer to one of the subunits in *Tm*-PPase (Vidilaseris et al., 2019) (Figure 3A) thereby trapping an asymmetric structure, with RMSD/C_α_ values reaching 1.6 Å for some of the loops vs. a highest RMSD/C_α_ of 0.5 Å for the equivalent loops in the symmetrical IDP-bound structure (PDB: 5LZQ). The ATC dimer bound *via* the β1-2 loop (Q268, K269, Q277), loop 8-9 (Q348, D351, V352), and loop 12-13 (G528, P530, P531) (*Tm-*PPase numbering) (Figure 3B). The chief difference between the 5LZQ and 6QXA structures is the presence of ATC in the crystals. Due to the ATC, the β1-2 loop moves closer to loop 12-13 and away from the dimer interface to an angle similar to that seen in the resting and product bound structures, indicating closure of the exit channel. Loop 12-13 also experienced some slight movements, but TMH12 still moves downwards by about 2 Å compared to the resting state. In addition, the coordination of Na^+^ at the ionic gate changes from pentacoordination to tetracoordination and the ion is displaced by about 1.2 Å from the IDP-only structure (Figure 3C). Thus, the ATC-bound subunit appears to be trapped in a closed exit channel state and cannot perform hydrolysis, and the unbound subunit cannot undergo its catalytic cycle, due to the ATC-bound subunit restricting its motions (Figure 2Be,f).

#### Inter-subunit Communication

Artukka et al. (2018) demonstrated that the wild-type K^+^/K^12.46^ center was important for inter-subunit communication. Substrate inhibition only occurred in K^+^ independent mPPases when the native K^12.46^ residue was present. In the K^12.46^A substituted enzymes there was no inhibition. Similarly, K^+^ dependent mPPases were not inhibited by excess PP_i_ unless K^+^ was present, even in A^12.46^K substituted enzymes. Consequently, this suggests the lysine and potassium ion are unable to perfectly replace each other, despite the findings of previous modeling studies (Kellosalo et al., 2012). This is further evidence that inter-subunit communication in response to excess PP_i_ is modulated by the native K^+^/K^12.46^ center, as its presence is required for excess substrate inhibition.

The recent asymmetrical ATC structure (see above) demonstrated direct linkage of TMH 12 to the dimer interface (Vidilaseris et al., 2019). Vidilaseris et al. suggest that, in the presence of K^+^, the movement of TMH 12 upon substrate binding to one subunit induces conformational changes in the dimer interface, which are then propagated to the other subunit. Moreover, when residues close to A^12.46^ were mutated to alanine, proton pumping and hydrolysis were uncoupled (Asaoka et al., 2014). This may be because this region is implicated in inter-subunit communication for cation translocation at normal PP_i_ levels, and thus when it is mutated, cation pumping ceased. This is further supported by the fact that mPPases are dimers, even though all the required catalytic domains are contained within a single monomer.

Early studies of the functional unit size of mPPases demonstrated that mPPases had to be functional dimers for cation pumping (Sarafian et al., 1992) but a single catalytic subunit was sufficient for hydrolysis (Sarafian et al., 1992; Tzeng et al., 1996). Although these studies were limited by the utilization of radiation-induced destruction, a technique which damages the entire molecule, not just specific sites (Kempner, 1993), they are still relevant when interpreting more recent evidence of subunit interdependence.

## Conclusion and Future Outlook

mPPases represent an important area of research, due to their essential functions in plants and human parasites. These functions make the enzymes both clinically and agriculturally relevant targets for modification. They are validated drug targets against a number of human diseases, and there is a wealth of evidence that manipulation of these proteins in plants can improve their tolerance to environmental stressors. An essential part of developing these modulation strategies is understanding the mPPase catalytic and cation pumping mechanism on a molecular level. Despite the recent structural and functional studies (Artukka et al., 2018; Vidilaseris et al., 2019), the mechanism involving both protein subunits remains elusive. To elucidate their full mechanism, further studies that couple time-resolved experimental techniques with molecular dynamics simulations are required.

## Author Contributions

AH, AG, and AK: conceptualization, writing-review, and editing. AG and AK: funding acquisition and supervision. AH: investigation and writing-original draft preparation.

### Conflict of Interest

The authors declare that the research was conducted in the absence of any commercial or financial relationships that could be construed as a potential conflict of interest.
